# Study of Optical Information Recording Mechanism Based on Localized Surface Plasmon Resonance with Au Nanoparticles Array Deposited Media and Ridge-Type Nanoaperture

**DOI:** 10.3390/nano12081350

**Published:** 2022-04-14

**Authors:** Sung-Mook Kang

**Affiliations:** School of Electronic and Electrical Engineering, Daegu Catholic University, Hayangro 13-13, Hayang-eup, Gyeongsan-si 38430, Gyeongbuk, Korea; kangsm@cu.ac.kr

**Keywords:** ridge-type nanoaperture, nanoparticle, localized surface plasmon resonance, optical recording, Finite-Difference Time-Domain

## Abstract

To verify the possibility of multiple localized surface plasmon resonance based optical recording mechanism, the present study has demonstrated that an Au nanoparticles array deposited with media combined with a ridge-type nanoaperture can amplify the |***E***|^2^ intensity of the incident optical light transmitted into the media under specific conditions. Using a numerical Finite-Difference Time-Domain method, we found that the optical intensity amplification first occurred in the near-field region while penetrating the ridge-type nanoaperture, then the second optical amplification phenomenon was induced between the metal nanoparticles, and eventually, the excitation effect was transferred to the inside of the media. In a system consisting of a Gold (Au) NPs deposited media and nanoaperture, various parameters to increase the |***E***|^2^ intensity in the near-field region were studied. For an Au nanoparticle size (Cube) = 5 nm × 5 nm × 5 nm, an inter-particle space = 10 nm, and a gap (between nanoaperture and media) = 5 nm, the |***E***|^2^ intensity of a ridge-type nanoaperture with an Au nanoparticles array was found to be ~47% higher than the |***E***|^2^ intensity of a ridge-type nanoaperture without an Au nanoparticles array.

## 1. Introduction

When light passes through metal nanoparticles (NPs)/nanostructures, it causes large enhancement of the electric field near the surface of the particles. This phenomenon is known as localized surface plasmon resonance (LSPR) [1,2,3,4,5,6]. Several studies related to LSPR, such as nanoscale pattering (or lithography), solar cell technology, and biotechnology, have been reported to date and are still ongoing [7,8,9,10,11,12,13,14,15,16,17,18,19,20,21,22,23,24,25,26,27]. In biotechnology in particular, the conventional fluorescence method generally requires nanoparticle (NP)-labeling to collect bioinformation [28]. However, several disadvantages, such as chemical instability, environmental quantum yield, and the high cost of detection instruments are yet to be overcome. As an alternative to this problem, we reported a study of LSPR based on an optical recording mechanism using a Gold (Au) NPs array deposited phase-change recording layer [29].

In our previous study [29], the method proposed aimed to induce an optical amplification effect based on the LSPR phenomenon by placing metal NPs on the upper surface of the recording media and focusing an incident beam with an objective lens. Furthermore, we confirmed the optical power amplification effect under specific conditions. However, to improve the signal to noise ratio (SNR), a higher optical power needed to be focused on the information recording layer of the media. Therefore, it was necessary to investigate the amplification of the incident beam more strongly and obtain a small-sized optical spot. As a solution, we reviewed a nanoscale bowtie, H- (or I-), and C-shaped aperture that could induce high optical amplification by forming a small-sized optical spot, with size less than a wavelength in the near-field region [30,31,32,33,34,35,36,37]. (The C-shaped aperture has been described as a “ridge-type nanoaperture” in the literature, and we will refer to it by this name only.) Actually, a metallic plate with subwavelength apertures was proposed in order to achieve a high spatial resolution [38,39,40], however, it was difficult to realize high power transmission efficiencies due to the diffraction limit. Generally, since the transmission power of subwavelength circular apertures exponentially decays with the fourth power of the aperture size [41], high transmission power through a small-sized aperture is an important requirement for optical information recording using Au NPs array deposited media. It can meet the optical high power transmission efficiency, as well as nanoscale small-sized optical spot, with a subwavelength aperture that has an extraordinary geometry.

**Figure 1:**
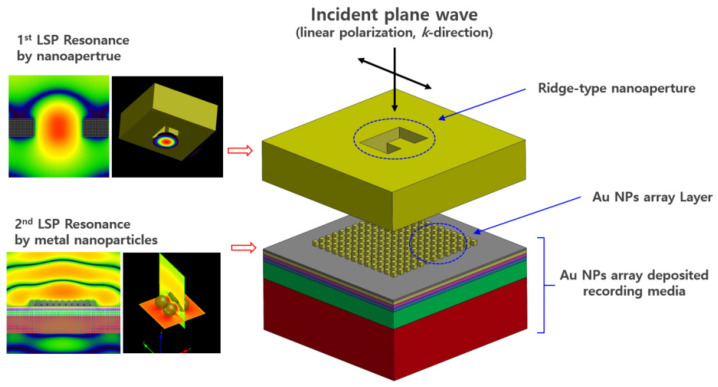
Schematic diagram of optical high power transmission into the Au NPs array deposited media with a ridge-type nanoaperture.

In the present study to obtain high power optical transmission and nanoscale optical spot based on LSPR for optical information recording in the near-field region, we synthesized a novel structure, in which there was a ridge-type nanoaperture and Au NPs array deposited media (see Figure 1). The incident beam was amplified by LSPR in the nanoaperture, which was again amplified by LSPR in the Au NPs array, and eventually the strongly enhanced optical power was transmitted through the media. (We will describe this phenomenon as LSP multiple excitations from this point onwards.) To analyze the proposed mechanism, various parameter studies, such as wavelength of the incident light, size of the cube type Au NPs, density of the Au NPs array, gap between the ridge-type nanoaperture and media, polarization dependence, and spot size due to beam skewness, were performed through the Finite-Difference Time-Domain (FDTD) method. The difference between the previous study and the current study is shown in Table 1.

## 2. Modeling and Condition

We used a numerical FDTD [42,43] simulation (XFDTD 6.5, Remcom [43]) to analyze the electromagnetic field behavior in an Au NPs array deposited on a disk surface and a ridge-type nanoaperture. The discretized Maxwell curl equation was calculated in the near-field region, and the stability condition related to spatial and temporal step size is expressed as:

(1) $$\Delta t=\frac{1}{c}{\left[\frac{1}{\Delta x^{2}}+\frac{1}{\Delta y^{2}}+\frac{1}{\Delta z^{2}}\right]}^{-1/2}$$

where *c* represents the velocity of optical light, and Δ*x*, ∆*y*, and ∆*z* indicate the spatial discretization in *x*, *y*, and *z* directions, respectively. The electromagnetic fields were computed for each cell in both space and time for each time step until steady state was attained. For the input sinusoidal source, the steady state was attained when all the scattered electromagnetic fields varied sinusoidally in time.

**Figure 2:**
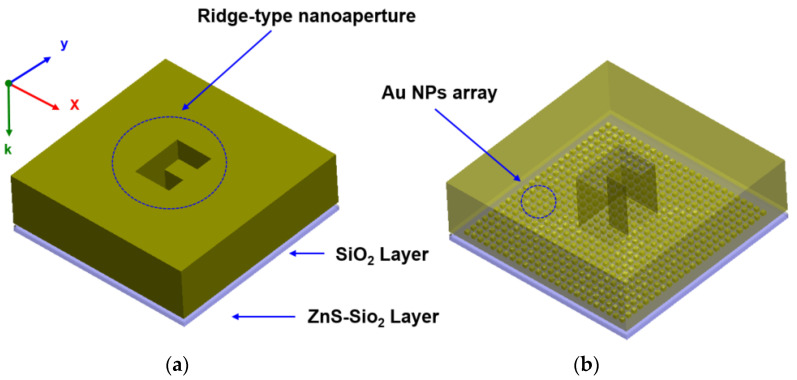
The structural FDTD model of Au NPs array deposited media with ridge-type nanoaperture (**a**) solid view, and (**b**) transparent view.

**Figure 3:**
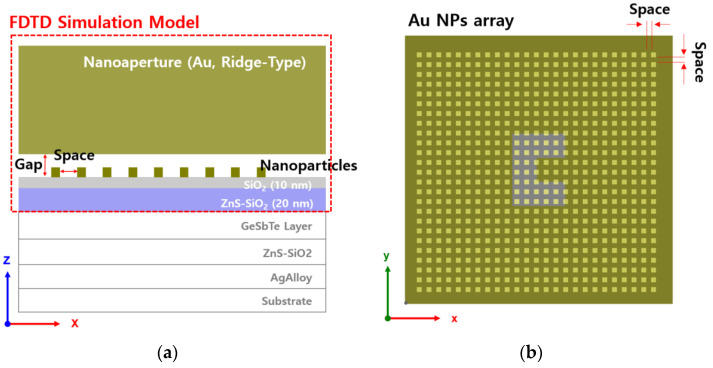
Au NPs array deposited media with ridge-type nanoaperture: (**a**) in the *y-z* plane; (**b**) in the *x-y* plane.

The purpose of this study is to examine the effect of optical intensity amplification when a high-power incident beam, before being passed through a media, was first amplified from a metal nanoaperture and then amplified again by an Au NPs array. The FDTD model used to verify the performance of LSP multiple excitations is shown in Figure 2. Figure 2a shows a 3D-view of the analysis model, in which the ridge-type nanoaperture and the Au NPs array structure are located on the upper surface of the media composed of ZnS–SiO_2_ and SiO_2_. For the ridge-type nanoaperture, the geometry that has already been verified [44] was used. Figure 2b shows the arrangement of the Au NPs array in the two-dimensional area between the ridge-type nanoaperture and the media. In general, the optical information recording media, a phase-change (GeSbTe) layer is located on the lower surface of ZnS–SiO_2_ and optical information is recorded there. In our structure, after the incident light has transmitted through the ridge-type nanoaperture and has been amplified in the near-field region, the enhanced electric field passes through the Au NPs and becomes amplified again. At the end, the multiple amplified electric field in the ridge-type nanoaperture and Au NPs array passes through SiO_2_ and ZnS–SiO_2_, and the obtained enhanced strong optical spot then reaches the phase-change layer region. Therefore, the FDTD analysis model was designed for the ridge-type nanoaperture, Au NPs array layer, SiO_2_ layer, and ZnS–SiO_2_ layers. Figure 3a shows the *x-z* view of the analysis model. The layer of the Au NPs array was located on the upper surface of the medium, which had a similar structure for optical information recording. Figure 3b shows the *x-y* view.

Several previous studies have already reported that an incident polarized light passing through a nanoscale ridge-type nanoaperture or metal nanoparticle causes electric field enhancement via LSPR in the near-field region. Since the main purpose of this study is to examine the mechanism and parameters, we analyzed various parameters that cause optical amplification when an incident beam is transmitted into a structure composed of a ridge-type nanoaperture and an Au NPs array. The main parameters important for amplifications are: (1) wavelength of incident light; (2) gap between the ridge-type nanoaperture and Au NPs array; (3) density of the Au NPs array; (4) size of the Au NPs; and (5) the type of metal. To change the density of the Au NPs array, the space between the closely spaced metal nanoparticles was changed. Chemically stable gold was used for both ridge-type nanoaperture and NPs. The Au NPs were modeled by using cube-type particles. The shape of the actual NPs is more advantageous for realizing the spherical shape. However, since the FDTD mesh size was set to 5 nm × 5 nm × 5 nm, a cube type nanoscale bead was used for both 5 nm and 10 nm. Although designing spherical metal particles for a 1 nm mesh size is possible, it requires a lot of simulation time and very high computing power (PC performance) for FDTD analysis. To vary the density of the Au NPs array mono-film, the Au NPs arrays were arranged on the area of 1.1 µm × 1.1 µm on the top surface of the medium with various spaces between the Au NPs. The range of spaces was increased from 0 nm to 40 nm in 5 nm increments.

The incident electric fields of a plane wave propagating in the *k*-direction, with wavelengths of λ 405 nm, 658 nm, and 780 nm, were sequentially used according to λ. These three wavelengths of light are typically used commercially in recording optical information on the phase-change layer of the optical recording information device. Thus, the advantage of using these three wavelengths is that the newly developed system can be easily integrated at a lower cost into these three laser wavelengths, which are already used in commercial products. However, it was confirmed from the FDTD simulation results that the phenomenon of optical intensity ${\left|E\right|}^{2}$ amplification was relatively small at wavelengths 405 nm and 658 nm in the ridge-type nanoaperture. Therefore, the 780 nm wavelength was only used for the FDTD simulation for the structure, having the combination of the nanoaperture and Au NPs array deposited layer. (The detailed simulation results will be discussed in Section 3). The complex dielectric constants of Au [45] were approximated using the modified Debye model [42]. The modified Debye model was used to describe the frequency dependence of the complex relative permittivity, which is given by:

(2) $$\tilde{\varepsilon }\left(\omega \right)=\varepsilon_{\alpha }+\frac{\varepsilon_{s}-\varepsilon_{\alpha }}{1+i\omega \tau }+\frac{\sigma }{i\omega \varepsilon_{0}}$$

where *ε_s_* represents static permittivity, *ε_α_* is the infinite frequency permittivity, *τ* is the relaxation time, and *σ* is conductivity. To simplify the calculation, the visible-light absorption of ZnS–SiO_2_ [46] in the air was considered negligible, that is the extinction coefficient (*k*) = 0. The calculated parameters are summarized in Table 2 and Table 3.

## 3. Results and Discussion

### 3.1. Performance Verification of Ridge-Type Nanoaperture

To examine the phenomenon of optical intensity ${\left|E\right|}^{2}$ amplification by ridge-type nanoaperture and Au NPs arrangement, the performance of the metal ridge-type nanoaperture, with respect to wavelength change of the incident light, was first reviewed. Au was used for the ridge-type nanoaperture. The geometry and 300 nm thickness of the ridge-type nanoaperture used in the current study was obtained from the verified model already published [44]. The incident linearly-polarized plane wave of wavelengths 405 nm (Blu-ray), 658 nm (DVD), and 780 nm (CD) were used for performance verification and these wavelengths are commonly used in optical disk drives (ODD).

**Figure 4:**
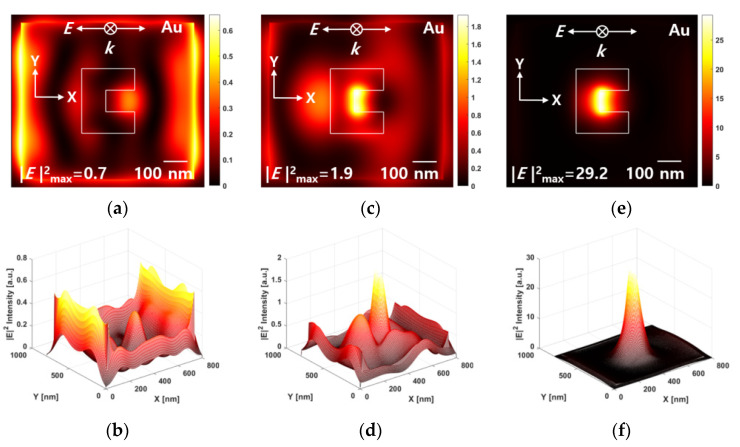
Near-field ${\left|E\right|}^{2}$ intensity distribution and beam profile in the *x-y* plane at a distance of 30 nm from the exit plane of ridge-type nanoaperture. (**a**,**b**) at λ = 405 nm. (**c**,**d**) at λ = 658 nm. (**e**,**f**) at λ = 780 nm.

Figure 4 shows the near-field ${\left|E\right|}^{2}$ intensity distribution and beam profile at 30 nm away from the ridge-type aperture. Since the cube-shaped Au NPs arrangement of size 5 nm and 10 nm were located between the metal ridge-type nanoaperture and the media, the near-field optical amplification phenomenon was investigated at a distance of 30 nm from the bottom surface of the nanoaperture. ${\left|E\right|}_{input}^{2}$ intensity of incident light is one. That is, ${\left|E\right|}^{2}$ intensity means ${\left|E\right|}_{output}^{2}/{\left|E\right|}_{input}^{2}$. As shown in the FDTD simulation results, the optical intensity, ${\left|E\right|}^{2}$ at a distance of 30 nm from the exit plane of the ridge-type nanoaperture at 405 nm wavelength was ~0.7, and eventually the ${\left|E\right|}^{2}$ optical intensity decreased by ~70% compared to the intensity of the incident beam (see Figure 4a,b. When the wavelength of the incident beam was 658 nm, the intensity ${\left|E\right|}^{2}$ was about ~1.9 at a point 30 nm away from the bottom of the aperture (see Figure 4c). However, on examining the optical profile, side lobes were found around the optical spot (see Figure 4d), which was due to the optical light amplification not being sufficient. For incident wavelength of 780 nm, ${\left|E\right|}^{2}$ intensity was ~30 times amplified at a distance of 30 nm from the exit plane of the nanoaperture (see Figure 4e). In other words, the transmitted ${\left|E\right|}^{2}$ intensity was more than ~30 times higher than the incident ${\left|E\right|}_{input}^{2}$ intensity. Furthermore, in the same range, the size of the optical spot, calculated from the FWHM, forms FWHM (*x* and *y*) = 95 nm × 140 nm, and it was confirmed that a normal optical spot profile was formed (see Figure 4f). From the analysis of the wavelength dependence of the ridge-type nanoaperture, it was confirmed that the wavelength of 780 nm was the most suitable for multiple LSPR based optical recording of the media, on which the layer of Au NPs array was deposited.

**Figure 5:**
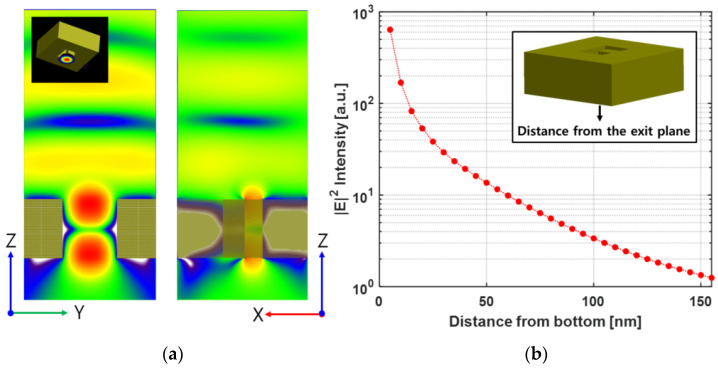
FDTD simulation results of the ridge-type nanoaperture: (**a**) ***E***-field distribution in the *z-y* plane and *x-z* plane; (**b**) ${\left|E\right|}^{2}$ intensity according to the distance from the exit plane.

When incident light measuring 780 nm in wavelength was passed through the ridge-type nanoaperture, an optical spot was formed and ${\left|E\right|}^{2}$ intensity amplification occurred due to the LSPR effect. These FDTD analysis results are shown in Figure 5a. So, it is necessary to know the effective range, in which the electric field is amplified from the exit plane of ridge-type nanoaperture. Figure 5b shows the intensity attenuation of ${\left|E\right|}^{2}$ from 5 nm to 150 nm at the bottom surface of the ridge-type nanoaperture. (The graph uses a log scale for just the *y*-axis.) At a point 5 nm away from the bottom surface of the nanoaperture, the optical ${\left|E\right|}^{2}$ intensity amplification was about 630 times. At a distance of 40 nm, the optical intensity amplification effect was about 20 times, and the optical intensity amplification effect was about three times at distance 100 nm. The effect of optical ${\left|E\right|}^{2}$ intensity amplification was negligible at 150 nm away. As a result, the ${\left|E\right|}^{2}$ intensity decayed exponentially rapidly as the distance increased from the bottom surface. Therefore, it is important to place the Au NPs array within the effective range of the electric field amplified from the nanoaperture. The structure should be designed in such a way that the amplified ***E***-field passes through the SiO_2_ layer and ZnS-SiO_2_ layer and is located at the position of the phase-change layer. In the present FDTD simulation model, the thickness of the SiO_2_ layer was designed to be 10 nm, and that of the ZnS-SiO_2_ layer to be 20 nm. At the end, that the phase-change layer was considered at a distance of 30 nm from the surface of the recording media. (There is no phase-change layer in this FDTD simulation, and the calculated results of the ***E***-field enhancement were analyzed at the corresponding position).

### 3.2. Nanopaterture and Au NPs Deposited Mdeia

This section presents the results of the FDTD analysis of the structure where a ridge-type nanoaperture was combined with an Au NPs array deposited media using an incident linear polarized plane wave, having a wavelength of 780 nm (${\left|E\right|}_{input}^{2}=1$). 

**Figure 6:**
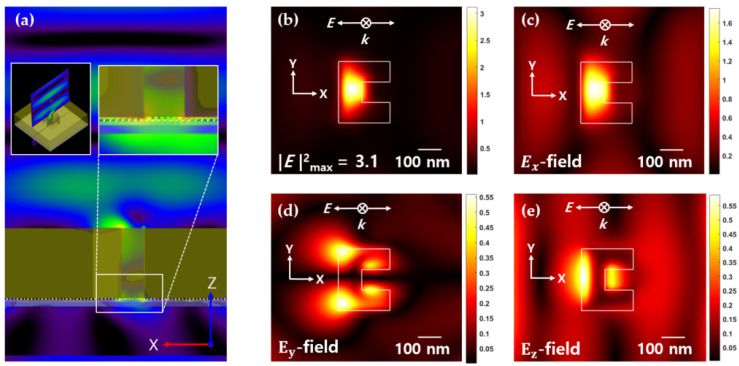
FDTD simulation results of the ridge-type nanoaperture with the Au NPs array deposited media from the bottom of the ZnS-SiO_2_ layer. (Cube-type size of NP = 5 nm × 5 nm × 5 nm, Gap = 5 nm, Space = 10 nm): (**a**) ***E***-field distribution in the *x-z* plane; (**b**) ${\left|E\right|}^{2}$ in the *x-y* plane; (**c**) $E_{x}$ in the *x-y* plane; (**d**) $E_{y}$ in the *x-y* plane; (**e**) $E_{z}$ in the *x-y* plane.

**Figure 7:**
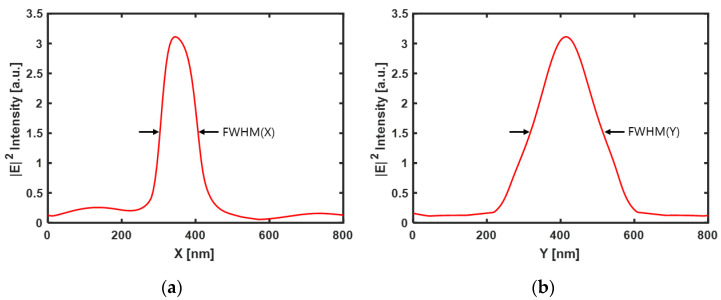
Maximum ${\left|E\right|}^{2}$ intensity profile at the cross-sections of the ridge-type nanoaperture with Au NPs array deposited media at the bottom surface of the ZnS-SiO_2_ layer. The spot size, calculated at FWHM, is (**a**) 100 nm × (**b**) 185 nm.

The *x-z* plane of the FDTD simulation result for a cube-type Au bead of dimension 5 nm × 5 nm × 5 nm, 10 nm space, and 5 nm gap is shown in Figure 6a, where *x*-polarized light (*k*-direction) is incident on the media. Figure 6b–e illustrate the computed contours of the components of the electric field distributions at the bottom surface of the ZnS-SiO_2_ layer. The calculated FWHM of the spot size was 100 nm × 185 nm (see Figure 7). Although there is a difference in the optical spot size and ${\left|E\right|}^{2}$ light intensity depending on the simulation conditions (space, gap, cube size), it was confirmed that similar electric field distributions were also formed for changes in the cube size (5 nm × 5 nm × 5 nm and 10 nm × 10 nm × 10 nm, respectively), space (0 nm~40 nm), and gap (5 nm~35 nm) of Au NPs (not shown here). The space range of distances was increased from 0 nm to 40 nm in 5 nm increments and the distance was increased from 5 nm to 35 nm in 5 nm increments.

**Figure 8:**
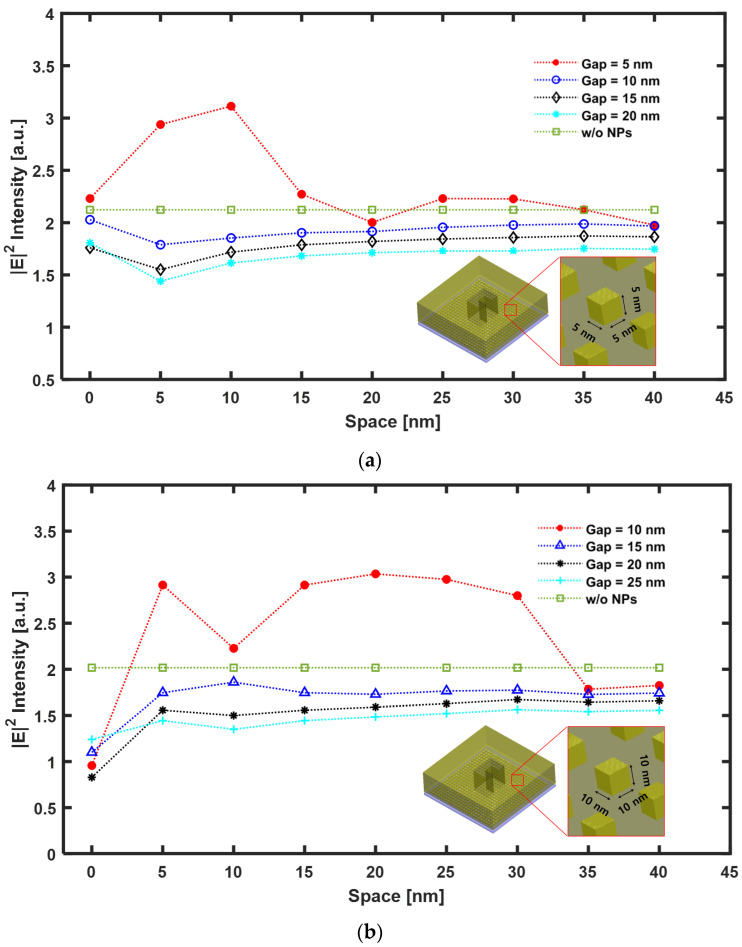
The calculated maximum ${\left|E\right|}^{2}$ intensity from the bottom surface of the ZnS-SiO_2_ layer: (**a**) The size of Au NP was 5 nm × 5 nm × 5 nm; (**b**) The size of Au NP was 10 nm × 10 nm × 10 nm.

Now, we will discuss the effect of optical amplification on the density change of the mono layer Au NPs array according to the change in space between the Au NPs. This signifies a change in the density of the Au NPs. Figure 8a shows the maximum ${\left|E\right|}^{2}$ calculated at the bottom of the ZnS-SiO_2_ layer, as it changed from 0 nm to 40 nm with an interval of 5 nm between the NPs. To verify the effective area of the ***E***-Field amplified by the LSPR effect formed through the nanoaperture, the $\mathrm{peak}\;{\left|E\right|}^{2}$ intensity is shown by changing the gap between the nanoaperture and media.

Figure 8a shows the results of the analysis of the Au NP cube size of 5 nm × 5 nm× 5 nm as a function of the variation in space between the Au NPs and the gap between the ridge-type nanoaperture and Au NPs array deposited media. Figure 8b shows the case where the Au NP cube size is 10 nm × 10 nm× 10 nm. For the structure where a ridge-type aperture was combined with Au NPs array, all of them displayed a similar tendency. There are two main points to this. First, although contact (space = 0 nm) between adjacent Au NPs cannot transmit the incident light to the media, a near-field interaction between the closely spaced Au NPs induces a strong ***E***-field enhancement based on LSPR. Second, since the LSPR has only the ***E***-field amplification effect in the near-field region, the closer the distance between the media and the nanoaperture, the greater the amplification effect will be. For cubes of sizes 5 nm × 5 nm × 5 nm and 10 nm × 10 nm × 10 nm, the ***E***-field amplification effect tended to disappear as the distance between the adjacent Au NPs increased. In Figure 8a, when the Au NPs were spaced at 10 nm and Gap = 5 nm, the maximum ${\left|E\right|}^{2}$ intensity was ~210% higher than the incident light intensity (${\left|E\right|}_{input}^{2}=1$) and 47% higher than that without any Au NPs array. Furthermore, this ${\left|E\right|}^{2}$ intensity amplification disappeared as the distance between the adjacent Au NPs increased by more than 15 nm. When the gap between the ridge-type nanoaperture and Au NPs array deposited media was farther away, no amplification effect of ${\left|E\right|}^{2}$ intensity could be observed. This is due to the ***E***-field enhancement phenomenon being induced from the ridge-type nanoaperture that exists only in the near-field region. 

Figure 8b shows the analysis result when sizes of the Au NP were 10 nm × 10 nm × 10 nm, and 5 nm × 5 nm × 5 nm. Although the amplification phenomenon is relatively small when the space is 0 nm, the maximum ${\left|E\right|}^{2}$ intensity increased in the range 5 nm to 30 nm of the space between the adjacent Au NPs. In particular, when the Au NPs were spaced at 20 nm and Gap = 10 nm, the maximum ${\left|E\right|}^{2}$ intensity attained was ~200% higher than the incident light intensity (${\left|E\right|}_{input}^{2}=1$), and it is 51% higher than that of the case without any Au NPs array. When the distance between the adjacent Au NPs was 0 nm, the maximum ${\left|E\right|}^{2}$ intensity was about −50% compared to the case without the Au NPs array. The reason for this is that since the increased nanoparticle size was the same as the increased thickness, the transmission of light decreased. It was confirmed that when the gap increased by 15 nm or more, the strongly amplified ***E***-field did not penetrate into the media. This signified that the Au NPs array should be located within the near-field enhancement region induced by the ridge-type nanoaperture. In addition, this showed that the gap between the ridge-type nanoaperture and the Au NPs layer plays an important role.

In summary, in structures comprising a ridge-type nanoaperture and an Au NPs array, since Au NPs with space = 0 nm function like a thin film, the transmitted light decreased, so that the maximum ${\left|E\right|}^{2}$ intensity also decreased inside the media. When the density of the Au NPs was high, the ***E***-field enhanced from the ridge-type nanoaperture was amplified again by the Au NPs array, so that the maximum ${\left|E\right|}^{2}$ intensity was increased inside the media. However, it was also confirmed that the optical amplification effect disappeared as the space was located relatively farther away. In addition, since the ***E***-field amplified by the ridge-type nanoaperture occurred only in the near-field region, it was confirmed that the Au NPs array should exist in the amplified near-field region. These FDTD calculation results showed that Au NP size, Au NPs array density, and gap between a ridge-type nanoaperture and NPs array layer are important design parameters.

### 3.3. Polarization and Beam Skewness

**Figure 9:**
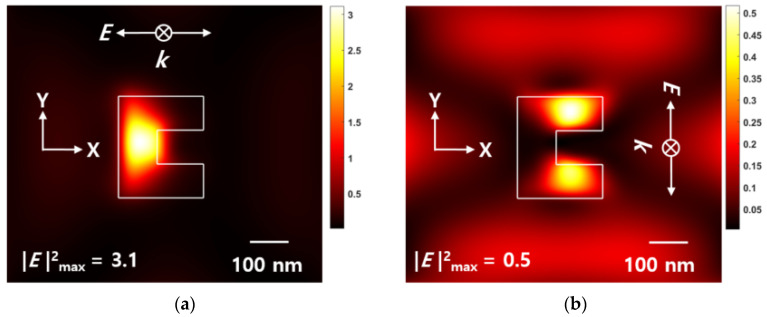
Electric field intensity${\left|E\right|}^{2}$ distribution in the *x-y* plane of ridge-type nanoaperture with Au NPs array deposited media from the bottom of the ZnS-SiO_2_ layer: (**a**) *x*-polarized light; (**b**) *y*-polarized light.

In this section, the effects of polarization and beam skewness were reviewed for the structure having the combination of a ridge-type nanoaperture and an Au NPs array. Figure 9 shows the near-field ${\left|E\right|}^{2}$ intensity field distribution of a ridge-type nanoaperture with Au NPs array, with respect to polarization. 

**Figure 10:**
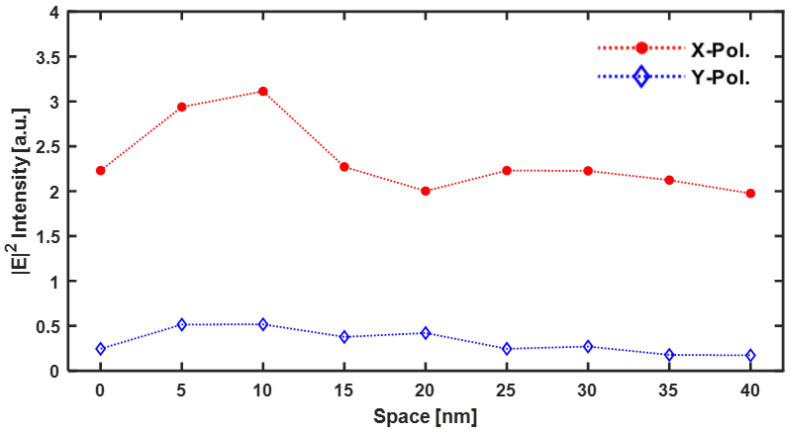
Difference in the maximum ${\left|E\right|}^{2}$ intensity from the bottom surface of ZnS-SiO_2_ layer due to polarization dependence.

Figure 9a shows the result of *x*-polarized light passing through a ridge-type nanoaperture, and Figure 9b shows the results for the polarization in *y*-direction. In this case, the size of Au NPs was 5 nm and the space was 5 nm. For *x*-polarized light, the ${\left|E\right|}^{2}$ intensity of the transmitted beam was ~three times higher than that of the incident beam (${\left|E\right|}_{input}^{2}=1$), and an elliptical optical spot was formed (see Figure 9a). By contrast, for y-polarized light, the ${\left|E\right|}^{2}$ intensity of the transmitted beam was ~0.5 times less than that of the incident beam (${\left|E\right|}_{input}^{2}=1$), and no normal optical spot was formed (see Figure 9b). Such observations can be explained by the fact that the surface plasmon focused on the ridge of the nanoaperture resonates due to *x*-polarization, so that the amplified ***E***-field passes through the space of the AuNPs and is amplified again. As the space of Au NPs increased, the result of the maximum ${\left|E\right|}^{2}$ intensity is shown in Figure 10. According to the simulation results, the ${\left|E\right|}^{2}$ intensity showed different effects depending upon the polarization. For *x*-polarized light, the ${\left|E\right|}^{2}$ intensity amplification occurred within a certain range. However, for the y-polarized light, ${\left|E\right|}^{2}$ intensity amplification did not occur over the entire range. Since no ${\left|E\right|}^{2}$ intensity amplification occurred, for the y-polarized light, from the ridge-type nanoaperture, the ${\left|E\right|}^{2}$ intensity amplification phenomenon could not be found on the exit plane of the ZnS-SiO_2_ layer. These results thus show that optical amplification phenomenon is possible in Au NPs-coupled media with a ridge-type nanoaperture, when suitable linear polarization that can amplify the ***E***-field in the ridge-type nanoaperture is used.

**Figure 11:**
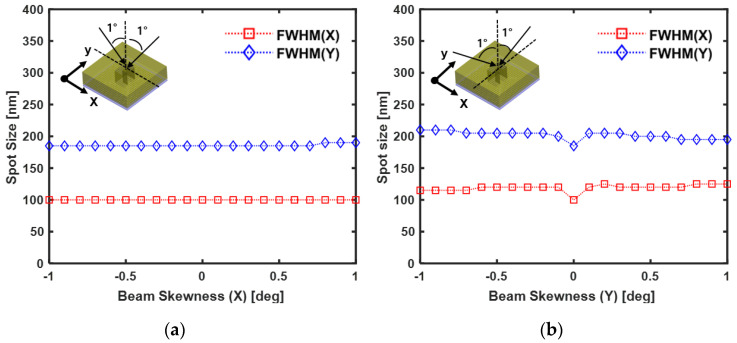
The calculated FWHM spot size on the bottom surface of the ZnS-SiO_2_ layer due to beam skewness: (**a**) *x*-axis beam skewness (−1°~+1°); (**b**) *y*-axis beam skewness (−1°~+1°).

Next, we will discuss the analysis results of beam skewness. In general, with regard to technology of optical information recording, an objective lens is assembled to a voice coil motor (VCM) actuator. An optical spot that penetrates the objective lens is focused on the phase-change layer. Then, the phase-change layer is changed from an amorphous state to a crystalline state, and this phase-change causes a difference in the optical reflection signal for detecting information. Since the objective lens and the VCM actuator are connected to the hinge wire, it has a structure, in which tilt occurs when the actuator moves in the focus and tracking direction toward the target point. Therefore, when the incident beam has beam skewness in the *x*- and the *y*-directions, the spot size needs to be calculated. The condition under which beam skewness is used is that it changes at intervals of 0.2 degrees, between −1° and +1° in the x- and y-directions. Figure 11 shows the analysis results of the bottom surface of the ZnS-SiO_2_ layer due to the beam skewness. Figure 11a shows the spot size calculation when skewness of the incident beam occurs in the *x*-axis direction. In the range (−1°~+1°) where beam skewness occurs, the calculated spot size shows FWHM (x) = 100 nm and FWHM (y) = 185 nm. Figure 11b shows the analysis results of the *y*-direction beam skewness of the incident beam. When the beam skewness is zero degrees, FWHM (x) = 100 nm and FWHM (y) = 185 nm. After beam skewness +/−0.1°, the calculated spot size was FWHM (x) = ~120 nm, FWHM (y) = ~200 nm, and no significant change was observed after beam skewness +/−0.1°. The reason for this is that the enhanced ***E***-field based on the LSPR effect was generated in a hemispherical shape in the near-field region of the nanoaperture bottom surface. Therefore, when using the nanoaperture, the tilt margin was secured. These analysis results thus provide important information about the experimental system.

## 4. Conclusions

The main goal of the present study was to analyze the LSPR based ${\left|E\right|}^{2}$ intensity amplification mechanism in optical recording for a structure having the combination of a ridge-type nanoaperture and an Au NPs array by using the FDTD calculation. At the laser wavelength used in general ODD optical pickup, it was confirmed that the phenomenon of LSPR based near-field optical amplification was the highest when suitable linear polarized light of a 780 nm wavelength was incident on the ridge-type nanoaperture. Furthermore, the incident ***E***-field was re-amplified when the amplified ***E***-field induced by the ridge-type nanoaperture penetrated into the Au NPs array under a specific structural condition. Such amplification occurred when the density of the Au NPs array was high, however, this disappeared when the density of the NPs was low. Under certain conditions, after the incident beam passed through the ZnS-SiO_2_ layer inside the media, the ${\left|E\right|}^{2}$ intensity was amplified by ~210%. At this time, the ${\left|E\right|}^{2}$ intensity was ~47% higher than the ${\left|E\right|}^{2}$ intensity without the Au NPs array.

In conclusion, the Au NPs array deposited media combined with a ridge-type nanoaperture can amplify ${\left|E\right|}^{2}$ intensity of the optical beam transmitted into the media under specific conditions. This preliminary study demonstrates the possibility of a cheap and robust information recording/reproducing mechanism, especially in the field of biochip technology that can record and reproduce bioinformation using metal nanoparticles.

## Figures and Tables

**Table 1:** Previous study and current study.

	Previous Study	Current Study
Structure	NPs	NPs and Nanoaperture
Mechanism	LSP Resonance	Multiple LSP excitations
Beam	Focused beam	Plane wave
Wavelength	405 nm, 658 nm, 780 nm	780 nm
Efficiency	High	Very High
Resolution	~λ/NA	FWHM (100 nm × 185 nm)

**Table 2:** Numerical value of the modified-Debye model for Au (Gold).

*λ* [nm]	Conductivity [S/m]	Relative Permittivity [Infinite Freq.]	Relaxation Time [s]	Static Permittivity
405	294,500	1	6.9385 × 10^−17^	−1.3078
658	3,162,200	1	3.7520 × 10^−15^	−1339
780	7,854,100	1	6.0315 × 10^−15^	−5349.2

**Table 3:** Numerical values of the modified-Debye model for SiO_2_ and ZnS–SiO_2_ (λ = 780 nm).

Material	Conductivity [S/m]	Relative Permittivity [Infinite Freq.]	Relaxation Time [s]	Static Permittivity
SiO_2_	785,980	1	−3.4189 × 10^−16^	31.3487
ZnS–SiO_2_	15,383,000	1	6.8779 × 10^−15^	−11,948
